# Comment on "Digital Mental Health and COVID-19: Using Technology Today to Accelerate the Curve on Access and Quality Tomorrow"

**DOI:** 10.2196/23023

**Published:** 2020-08-21

**Authors:** Nikhil Jain, Mahesh Jayaram

**Affiliations:** 1 Northwestern Mental Health Melbourne Health Melbourne Australia

**Keywords:** telepsychiatry, COVID-19, Australia

The article titled, “Digital Mental Health and COVID-19: Using Technology Today to Accelerate the Curve on Access and Quality Tomorrow” [1] was an interesting read. The coronavirus disease (COVID-19) pandemic has catalyzed the use of telepsychiatry and broken down barriers, giving way to a “can do” approach. The editorial rightly discusses accelerating the use of information technology in psychiatry. This is a good time to collect global experiences on this subject; we discuss telepsychiatry use in metropolitan Melbourne, Australia.

Although telepsychiatry has always been available, there was a notable reluctance to embrace it despite support from accrediting bodies. The Royal Australian and New Zealand College of Psychiatrists, for example, endorsed its implementation in situations where in-person consults are not feasible. Like most public mental health services in Australia, our service also transitioned to telepsychiatry quickly during the COVID-19 pandemic to provide safe consulting services. We collated some of our experiences by conducting a rapid survey, which was sent out to 40 professionals, and received 14 responses. In total, 70% of the respondents reported that teleconsults had increased significantly during this time. A further 70% noted that there were fewer missed appointments, and the workflow was generally more efficient following the transition to telepsychiatry; 79% were satisfied with the care they were able to provide.

A few challenges were also encountered during this transition. Difficulties using new technology were experienced by 70% of the respondents. A plethora of platforms, such as Zoom, Doxy, CoViu, etc, are available in Australia, each with its own benefits and shortcomings. There was a lack of consensus about which platform to use and guidelines changed frequently, which required learning and relearning when switching between platforms; this created difficulty for some staff and patients alike. Another challenge experienced by the team was maintaining therapeutic alliance (eg, effective communication of empathy and use of nonverbal gestures during a teleconsult). The lack of data available for video consults for some disadvantaged patients was also noted. Training around the effective use of information technology could be helpful, and the survey revealed that only 1 out of 14 respondents had sought such training.

Suitability of patients for teleconsults is a key issue to consider. Often, patients with anxiety and mood spectrum disorders were easier to engage compared to patients with psychosis or drug use. There are advantages of using video over telephone since someone who is distressed (eg, crying) or hypomanic cannot be easily identified through the latter.

Another challenge was the absence of a seamless delivery method to ensure electronic prescription dispatch to the pharmacy. Faxing or emailing prescriptions not only increased workload but was also unreliable. We hope that adding the Australian experience of using telepsychiatry during the COVID-19 pandemic will help in planning better telepsychiatry services in the future—a future where the integration of key clinical functions can occur within telehealth platforms that enable the delivery of accessible, seamless, and safe care.

## Abbreviations

COVID-19: coronavirus disease
